# Immunological consequences of intermittent preventive treatment against malaria in Senegalese preschool children

**DOI:** 10.1186/1475-2875-9-363

**Published:** 2010-12-17

**Authors:** Denis Boulanger, Jean Biram Sarr, Florie Fillol, Cheikh Sokhna, Badara Cisse, Anne-Marie Schacht, Jean-François Trape, Gilles Riveau, François Simondon, Brian Greenwood, Franck Remoué

**Affiliations:** 1 Unité Mixte de Recherche 145, Institut de Recherche pour le Développement and Université Montpellier 1, 911 avenue Agropolis, BP64501, 34394 Montpellier, France; 2 ONG Espoir Pour La Santé (EPLS), BP 226, Saint-Louis, Sénégal; 3 IRD, UMR198, Route des Pères Maristes, BP 1386, 18524 Dakar, Sénégal; 4 Université Cheikh Anta Diop, Département de Parasitologie, Dakar, Sénégal; 5 Immunology and Infection Center of Lille, Pasteur Institute of Lille, France; 6 IRD-UR024, Montpellier, France; 7 London School of Hygiene & Tropical Medicine, London, United-Kingdom; 8 IRD, UR016, 911 avenue Agropolis, BP64501, 34394 Montpellier, France

## Abstract

**Background:**

Intermittent preventive treatment in children (IPTc) is a promising strategy to control malaria morbidity. A significant concern is whether IPTc increases children's susceptibility to subsequent malaria infection by altering their anti-*Plasmodium *acquired immunity.

**Methods:**

To investigate this concern, IgG antibody (Ab) responses to *Plasmodium falciparum *schizont extract were measured in Senegalese children (6 months-5 years old) who had received three rounds of IPTc with artesunate + sulphadoxine-pyrimethamine (or placebo) at monthly intervals eight months earlier. Potential confounding factors, such as asexual malaria parasitaemia and nutritional status were also evaluated.

**Results:**

Firstly, a bivariate analysis showed that children who had received IPTc had lower anti-*Plasmodium *IgG Ab levels than the non-treated controls. When epidemiological parameters were incorporated into a multivariate regression, gender, nutritional status and haemoglobin concentration did not have any significant influence. In contrast, parasitaemia, past malaria morbidity and increasing age were strongly associated with a higher specific IgG response.

**Conclusions:**

The intensity of the contacts with *P. falciparum *seems to represent the main factor influencing anti-schizont IgG responses. Previous IPTc does not seem to interfere with this parasite-dependent acquired humoral response eight months after the last drug administration.

## Background

Malaria elimination is now considered a realistic goal for an increasing number of countries [1]. It requires control of the infection in the most at-risk groups, namely pregnant women [2] and children [3]. The distribution of anti-malarial drugs at predetermined regular intervals (Intermittent Preventive Treatment, IPT) to individuals regardless of their malaria status, already implemented during pregnancy, is under clinical evaluation in infants (IPTi, reviewed in [4]), and in preschool (IPTc, [5-9]) and school-aged children (IPTsc, [7,10,11]). Seasonal IPTc (sIPTc) is defined as the administration of IPT to children during the transmission season in locations where malaria transmission is not perennial, mainly in the African Sahelian belt. IPT strategies raise several concerns which are under scrutiny, such as optimal schedule, acceptability, drug resistance, implementation, cost-efficiency, but one question requires an urgent answer-does IPT impair the development of specific immunity? IPT is able to clear a large number of circulating parasites, thus reducing the amount of contacts which are normally required to develop naturally acquired immunity to malaria [12].

Additionally, immuno-suppression has been reported from experimental studies of artemisinin-derivatives [13]. In the case of IPTi, some individual trials provided evidence that treated infants were subsequently, more susceptible to malaria or anaemia, the so-called "rebound effect" [14,15], but an overall analysis did not show any evidence of rebound [16]. Attention was given mainly to the possible interference between treatment and the infants' response to EPI vaccines, which are delivered at the same time [17-19]. However, only two studies considered specific anti-*Plasmodium *immune responses. In Mozambique, sulphadoxine-pyrimethamine (SP) given at the age of three, four, and nine months did not significantly modify the development of naturally acquired antibody (Ab) responses to several *Plasmodium falciparum *antigens up to 24 months of age [20]. On the other hand, in Ghana, anti-schizont Ab levels were significantly lower in children treated once with SP than in controls [21]. In the latter study, IgG levels were related to the frequency of past infections. Two IPTc trials conducted in children less than five years of age, have demonstrated a lack of a clinical rebound-effect one year after IPT delivery, using SP + artesunate in Senegal [6] and SP in Mali [7]. In Ghana [9], malaria incidence during the post-intervention period was increased by 62% in infants who received six monthly artesunate + amodiaquine, but this rebound was not seen in children aged one year or more at the time of drug administration. Immunological status, known to be closely age-dependent, was not assessed in any of these IPTc trials. Therefore, the objective of the present study was to check whether IPTc had any impact on the anti-*Plasmodium *IgG response in the Senegalese study [6], eight months after the last IPT delivery. To improve our understanding of the mode of action of IPT [4,22], epidemiological features of the study children were incorporated as potential confounding factors in a multivariate analysis.

## Methods

### Cohort follow-up

The study population came from the community of Niakhar, situated in Central Senegal, 145 km east from Dakar, where regular demographic surveillance has been maintained since 1963 [23]. It is an open savannah area, with less than 500 mm of rainfall per year. Malaria transmission is markedly seasonal and classified as mesoendemic, with most infections occurring between July and October and most clinical cases occuring in September-October. The average entomological inoculation rate is 10 infective bites per year with sharp variations between villages depending on their distance to the closest *Anopheles *larval breeding sites [24]. In 2002, a double-blind, randomized, placebo-controlled trial demonstrated, on an initial cohort of 1203 children (6 weeks to 5 years old), that a combination of artesunate and SP administered preventively on a monthly basis between September and November reduced the number of malaria attacks in treated children by 86% [6]. The active (weekly domiciliary visits) and passive (dispensaries) detections of malaria cases relied on clinical symptoms as previously defined [6] and used a parasite density of 3,000 *P. falciparum *asexual stage parasites/μl as the minimal threshold to consider a case as one of confirmed malaria. A cross-sectional survey was done in December 2002, in order to measure the impact of IPTc on the prevalence of children with parasitaemia, parasites resistant to sulphadoxine-pyrimethamine and anaemia at the end of the clinical follow-up period, and seven months later, in July 2003, before the beginning of the next rainy season. Anthropometric measurements were collected as previously described [25]. Capillary blood smears were made from a fingerprick sample, allowing the assessment of *Plasmodium *asexual blood stages parasitaemia. Haemoglobin concentrations were measured in a HemoCue Machine^® ^as previously described [6]. Sera were obtained by centrifugation then stored at -20°C until tested. From the 929 children who attended the cross-sectional survey in July 2003, a randomization list was computer generated and a subsample of 350 children was selected for serological screening. 12 blood samples could not be technically processed, leaving 338 available for assay.

### Ethics

Approval of the study was obtained from the ethical review committees of the Senegalese government, the French Institute of Research for Development, and the London School of Hygiene and Tropical Medicine. Informed consent was obtained from parents or guardians before any child was included in the study.

### Evaluation of anti-*Plasmodium *IgG levels by ELISA

IgG antibodies were screened using a biotin-avidin amplified indirect ELISA. *Plasmodium falciparum *schizont extract was obtained from infected erythrocytes and coated on flat-bottom microtiter plates (Nunc, Roskilde, Denmark) at a concentration of 2 μg/ml. After blocking the plates with gelatin 0.5% in PBS, the plasma samples and a mouse biotinylated mAb to human IgG (BD Pharmingen, San Diego CA, USA) were successively diluted in PBS-Tween 0.1% at 1:100 and 1:1000, respectively. After incubation with a peroxidase-conjugated streptavidin (Amersham Biosciences, les Ulis, France), ABTS (2.2'-azino-bis (3-ethylbenzthiazoline 6-sulfonic acid) diammonium, Sigma, St Louis, MO, USA) was added and optical density (OD) was measured at 405 nm. All sera were tested in duplicate wells coated with antigen together with a third single uncoated well to take into account non-specific bindings. A pool of positive sera was run in all the plates allowing adjustments for inter-plate variations. A negative pool (non-exposed European individuals) was also included in each plate.

### Statistical methods

Comparisons between medians used the Mann-Whitney test. Multivariate hierarchical linear regression was performed to identify variables independently associated with antibody measurements. Since these were not normally distributed, optical densities were log transformed. Potential covariates entered in the model were: the treatment group (IPT vs placebo), age (12-24; 24-36; 36-48; 48-60 and ≥ 60 months old at the time of blood sampling), sex, nutritional status (normal; wasted; stunted as previously described [26]) assessed in November 2002 and in July 2003, number of malaria attacks between September and December 2002 (continuous), presence of blood asexual stages of *P. falciparum *in December 2002 and in July 2003, haemoglobin concentration (g/dL) in July 2003 (continuous). Reference categories were as follows: females (gender), two years old class (age), normal (nutritional status), no malaria case (morbidity), no detected trophozoite (parasitaemia) and lowest value (haemoglobin). To account for the hierarchical structure of the data (groups of children sampled in 11 different villages), we included a random effect component at the village level in the regression model. Significance of the random effect was tested using a likelihood ratio test. Analyses were performed using the Stata software (Version 10.0; Stata Corp., College Station, TX). The significance limit was P < 0.05.

## Results

### Cohort description

The 338 sampled children (49.1% males) lived in 11 villages (8-66 per village) and 158 (46.7%) received IPT in 2002. In this sub-sample, the overall malaria incidence in 2002 was 0.406 (placebo) and 0.089 (IPT) cases/child over the 3.5 months follow-up. Villages differed significantly in terms of parasite rates, ranging from 0 to 43.2% in December 2002 and from 4.8 to 42.9% in July 2003 (P = 0.007 and 0.006, respectively).

### Influence of 2002 treatment on anti-*Plasmodium *IgG responses

#### Bivariate analysis

**Figure 1:**
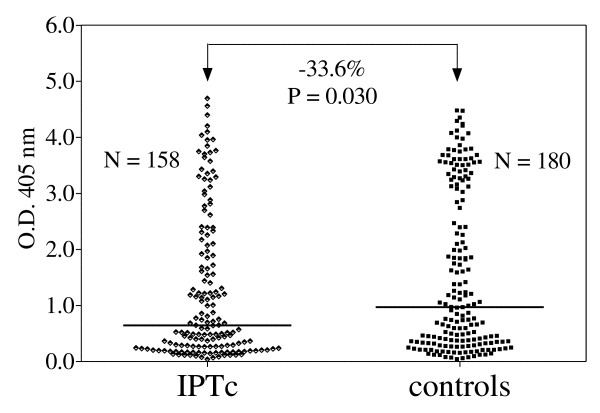
**IgG antibodies against *Plasmodium falciparum *schizont extract measured in July 2003**. Children received, in September-October-November 2002, either intermittent preventive treatment (IPTc) with artesunate + sulphadoxine-pyrimethamine or placebo (controls). Bars indicate the median value for each group. The two groups were compared using a bivariate Mann-Whitney test.

Levels of IgG Ab against *P. falciparum *schizont in sera collected in July 2003 from children who had received either IPT or a placebo the previous year are shown in Figure 1. The median antibody level in the IPTc group was significantly lower by one-third compared to the non-treated controls (Mann-Whitney, P < 0.05). Considering the heterogeneity of the O.D. distributions within each group, potential confounding factors were incorporated into a multivariate model.

#### Multivariate analysis of potential confounding epidemiological factors

**Figure 2:**
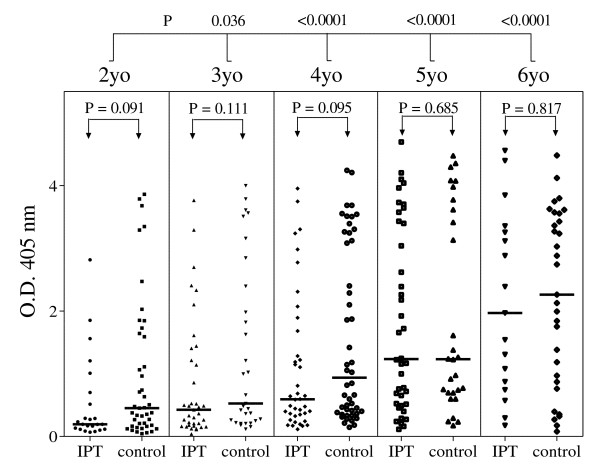
**Anti-*Plasmodium falciparum *schizont IgG levels in July 2003**. Anti-*Plasmodium falciparum *schizont IgG antibodies in 338 children presented according to their age class and to the treatment they received in September-October-November 2002 (IPT or placebo). Bars indicate the median value for each sub-group. P values drawn for comparisons between the age groups obtained using multivariate analysis (see Table 1) are displayed above the frame. P values obtained by comparing the IPT and control groups using bivariate analysis (Mann-Whitney test) are displayed within the frame. yo: years old

**Figure 3:**
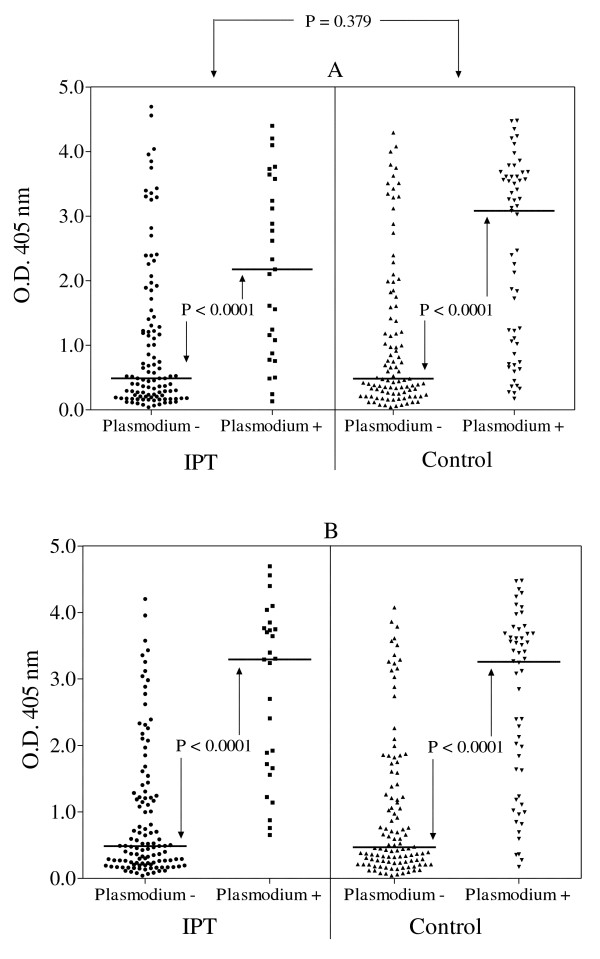
**Anti-*Plasmodium falciparum *schizont IgG levels in July 2003**. A comparison of anti-*Plasmodium falciparum *schizont IgG antibodies in 290 children surveyed in December 2002 (panel A) and in 305 children surveyed in July 2003 (panel B) by parasite status. All children had received IPT or placebo in September, October and November. The P value between the IPT and the control groups originates from a multivariate analysis (see Table 1) whereas Mann-Whitney tests were used to compare *Plasmodium *carriers to non-carriers. Bars indicate the median value for each sub-group.

Data were available from the 338 children except for information on parasitaemia for 48 children in December 2002 and 33 in July 2003 (Table 1). When covariates were introduced into the multivariate model, the 2002 treatment group, gender, nutritional status and haemoglobin concentration did not appear to have a significant influence on the IgG titres although stunting in July 2003 tended to be associated with a lower response without reaching statistical significance (P = 0.144). In contrast, age was positively and strongly associated with increasing anti-*Plasmodium *Ab levels (Figure 2), especially in the older children. Similarly, carriage of *Plasmodium *asexual stages, both at the end of the first transmission season (December 2002) and at the beginning of the following transmission season (July 2003), was associated with a high Ab response, irrespective of the treatment received in 2002. Median IgG Ab levels were 5.2 (CI95%: 3.7-7.1) and 6.8 (CI95%: 5.4-8.0) times higher in parasitaemic children compared to blood film negative children at both cross-sectional surveys (Figure 3). In addition, the number of malaria attacks experienced in 2002 was positively linked to anti-*Plasmodium *IgG levels (data not shown). Village of residence had a significant (P = 0.007) impact on antibody levels. The highest median specific IgG levels were observed in the villages closest to potential mosquito larval breeding sites (data not shown). No interaction was found between the covariates.

**Table 1:** Multivariate mixed-effect regression model of anti-schizont IgG by treatment group.

Variables	Period	Estimates	*P*-value	95% Confidence interval	
Treatment group	2002	0.112	0.379	-0.137	0.360
Gender	-	-0.031	0.787	-0.255	0.193
Age class					
3 years (y)	2003	0.410	0.036	0.027	0.793
4 y	2003	0.733	< 0.0001	0.375	1.091
5 y	2003	0.919	< 0.0001	0.530	1.308
6 y	2003	1.035	< 0.0001	0.631	1.439
Stunting	November 2002	0.043	0.814	-0.315	0.401
	July 2003	-0.253	0.144	-0.592	0.086
Wasting	November 2002	0.017	0.933	-0.393	0.428
	July 2003	-0.011	0.963	-0.492	0.469
Malaria morbidity	2002	0.243	0.034	0.018	0.468
Parasitaemia	December 2002	0.573	< 0.0001	0.308	0.839
	July 2003	0.931	< 0.0001	0.653	1.210
Haemoglobin	July 2003	0.003	0.936	-0.082	0.089
Constant term	-	-1.330	0.005	-2.257	-0.404
Random-effects parameters					
Village	-	0.244	-	0.114	0.520
Residual	-	0.884	-	0.807	0.969

Gender, age, nutritionnal status and malaria indicators were evaluated as potentially confounding factors, village as a random factor. P values were considered as significant when < 0.05.

## Discussion

Despite some debate on its efficacy, evidence is growing that IPT is a potentially valuable tool to control malaria in young children [27]. It may be particularly useful in areas with seasonal malaria transmission where drug delivery can be focused on a limited period of time when transmission and malaria risk are maximal. However, the potential negative effect of IPT on the development of natural acquired immunity against the parasite remains an important point to be investigated [28]. Theoretically, IPT could interfere with the anti-*Plasmodium *immune response via two main distinct mechanisms, either by a direct immunosuppressor effect of the drugs or, indirectly, by reducing contacts between the treated child and *Plasmodium *thus impairing immunity development. There is already evidence to support the latter mechanism in the case of weekly chemoprophylaxis [29] and this has led to concerns over its widespread use. Unlike some other classes of anti-malarial molecules, including the 4-aminoquinolones [30,31], neither SP or artesunate is thought to be immunosuppressive. Moreover, several IPTi trials (reviewed by [32]) have shown that SP and artesunate did not have an effect, positive or negative, on the infant response to various childhood vaccines (diphtheria, tetanus, pertussis, polio, hepatitis B, Hib, measles). The most likely mechanism by which IPTc reduces malaria morbidity combines therapeutic (clearing of existing infections) and preventive (allowing immunity development) effects, the prophylactic effect being predominant [33]. IPTi studies have shown that in areas where *P. falciparum *is still susceptible to SP, a single dose of SP provides infants with a protection lasting from 4-6 weeks [34] to 30-60 days [35] according to the setting, as might be anticipated from the half lives of the component drugs.

The present study showed that children less than five years who had received preventively the combination of SP + artesunate during the rainy season had a slightly lower level of anti-*Plasmodium *schizont Abs compared to non-treated control children at the beginning of the next transmission season, eight months later. These findings are in agreement with the reduced anti-schizont IgG response measured in Ghanaian infants six months after a single dose of SP given alone [21] although this study differed in several aspects, noticeably in the age of the participants (malaria-naïve infants in Ghana versus 1-6 years old children with some immunity in Senegal) and on the endemicity levels (perennial transmission in rural Ghana with 400 infective bites per person-year [36] versus seasonal transmission in Senegal with 10 infective bites per year [24]).

In a multivariate model, four co-variables significantly influenced anti-*Plasmodium *IgG levels: age, village, past malaria morbidity and *Plasmodium *carriage. Age was a major determinant of specific IgG titres, especially among children older than four years. This is in agreement with findings in Northern Senegal where anti-schizont IgG responses increased progressively in one to five-year old children and then remained stable up to nine years of age [37]. Age-associated maturation of the immune system, cumulative impact of mosquito infecting bites and production by the oldest children of long-lived plasma cells [38] may explain the natural influence of age. In accordance with what was observed in Mozambique [20] and in Ghana [21], children who experienced clinical attacks of malaria had higher anti-*Plasmodium *IgG responses in July 2003 than those who did not become sick. These observations suggest that release into the bloodstream of large numbers of schizont-derived *Plasmodium *stages during a malaria attack provides substantial antigenic stimulation to the immune effector cells. As reported in the two IPTi antibody surveys previously quoted [20,21], *Plasmodium *carriage at the time of blood sampling (July 2003) was associated with a strikingly higher anti-schizont IgG response. Increased humoral responses to various antigens in parasitized children compared to controls is a consistent observation [37,39], reflecting an increased exposure of the immune system to blood-stage parasites. Similarly, differences in the frequency of infectious anophelines bites [24] may account for the observed significant heterogeneity of specific IgG titres between villages. Surprisingly, parasite carriage seven months before the blood sampling was also strongly and positively linked to a high specific Ab response. Interestingly, parasite rates did not change significantly during the 2003 dry season suggesting that parasites can persist in humans from one transmission season to the other and elicit a steady production of specific Abs through continuous antigenic stimulation. Taken as a whole, it appears that the level of anti-*Plasmodium *schizont Abs at a given time reflects a cumulative process of specific immunoglobulin production positively related to the level of contact between the host immune system and blood-stage parasites. It underlines the fact that *Plasmodium *carriage is the key factor for anti-schizont IgG production, irrespective of the preventive treatment received, although the possibility of some contributory effect from the antimalarial drugs used for IPT cannot be completely excluded.

## Conclusions

To conclude, administration of SP and artesunate used as sIPTc slightly decreased the humoral reactivity to a crude *Plasmodium *extract. Specific IgG Abs appeared to increase with the amount of exposure to *Plasmodium *asexual stages at the time of blood sampling but also to exposure during the previous 10 months. A more accurate analysis of the immune response, looking at different putatively protective isotypes and antigens [40], would be valuable. Previous studies have shown, in The Gambia, that stopping chemoprophylaxis after a period of several years increased the risk of clinical malaria [41]. Monitoring the potential rebound effect in children who will receive IPTc for several consecutive years will also be of great interest [42].

## List of abbreviations used

Ab: Antibody; EPI: Expanded Program on Immunization; Hib: *Haemophilus influenzae *type b; IPT: Intermittent Preventive Treatment; IPTc: Intermittent Preventive Treatment in children; IPTi: Intermittent Preventive Treatment in infants; IPTsc: Intermittent Preventive Treatment in school-aged children; OD: Optical Density; PBS: Phosphate-Buffered Saline; sIPTc: seasonal Intermittent Preventive Treatment in children.SP: Sulphadoxine-Pyrimethamine.

## Competing interests

The authors declare that they have no competing interests.

## Authors' contributions

DB, BG and FR conceived the study design. CS, BC and JFT coordinated the cohort follow-up. JBS, AMS and FF performed the bench work. DB and FR analysed the data and drafted the manuscript. All the authors amended the draft and approved the final manuscript.
